# Diverse Profiles of Anxiety Related Disorders in Fragile X, Cornelia de Lange and Rubinstein–Taybi Syndromes

**DOI:** 10.1007/s10803-016-3015-y

**Published:** 2017-01-31

**Authors:** Hayley Crawford, Jane Waite, Chris Oliver

**Affiliations:** 1 Centre for Research in Psychology, Behaviour and Achievement, Coventry University, James Starley Building (JSG12), Priory Street, Coventry, CV1 5FB UK; 2 Cerebra Centre for Neurodevelopmental Disorders, School of Psychology, University of Birmingham, Edgbaston, Birmingham, B15 2TT UK

**Keywords:** Anxiety, Genetic syndromes, Fragile X syndrome, Cornelia de Lange syndrome, Rubinstein–Taybi syndrome, Intellectual disability

## Abstract

Anxiety disorders are heightened in specific genetic syndromes in comparison to intellectual disability of heterogeneous aetiology. In this study, we described and contrasted anxiety symptomatology in fragile X (FXS), Cornelia de Lange (CdLS) and Rubinstein–Taybi syndromes (RTS), and compared the symptomatology to normative data for typically-developing children and children diagnosed with an anxiety disorder. Scores did not differ between children diagnosed with an anxiety disorder and (a) participants with FXS on social phobia, panic/agoraphobia, physical injury fears, and obsessive–compulsive subscales (b) participants with CdLS on separation anxiety, generalized anxiety, panic/agoraphobia, physical injury fears and obsessive–compulsive subscales, and (c) participants with RTS on panic/agoraphobia and obsessive–compulsive subscales. The results highlight divergent profiles of anxiety symptomatology between these groups.

## Introduction

Anxiety is evident more often in individuals with intellectual disability than those of typical development. Prevalence rates for an anxiety disorder in children and adolescents with an intellectual disability range from 3 to 21.9% (Reardon et al. 2015; Royston et al. 2016). These prevalence rates are higher than those for the general population for which the prevalence rate for typically developing children and adolescents is 3 to 6.5% (Green et al. 2005; Polanczyk et al. 2015). DSM-5 lists 12 types of anxiety disorder (American Psychiatric Association 2013): separation anxiety disorder, selective mutism, specific phobia, social anxiety disorder, panic disorder, panic attack, agoraphobia, generalized anxiety disorder, substance-induced anxiety disorder, anxiety disorder due to another medical condition, other specified anxiety disorder and unspecified anxiety disorder.

Genetic syndromes can be associated with a heightened prevalence of particular characteristics (Dykens et al. 2000); for example, heightened levels of self-injurious behavior in Lesch–Nyhan syndrome, aggression in Angelman syndrome and excessive friendliness in Williams syndrome (see Waite et al. 2014 for a review). Furthermore, research has indicated that some genetic syndromes, such as Williams syndrome, 22q11.2 deletion, fragile X (FXS) and Cornelia de Lange syndrome (CdLS), are also at greater risk of anxiety compared to the general population (CdLS: Basile et al. 2007; FXS: Cordeiro et al. 2011; Williams syndrome: Dykens 2003; 22q deletion: Fung et al. 2010). These genetic syndromes are often associated with specific types of anxiety disorder. For example, anxiety in people with Williams syndrome is more likely to be generalized, or related to specific phobias and fears, than to social situations (Leyfer et al. 2009).

Anxiety has previously been investigated in FXS, CdLS and Rubinstein–Taybi syndromes (RTS), which form the focus of the current study. Although previous research has suggested a heightened risk of anxiety in these populations, this research has not explored the symptoms of different types of anxiety disorder using comparable assessment instruments. Identifying the symptomatology of different types of anxiety disorder most associated with these genetic syndromes will further understanding of the difficulties experienced by these groups. This will provide valuable information to clinicians, aiding them in developing early intervention strategies and targeted syndrome-sensitive interventions.

The aim of the present study is to delineate the profile of anxiety symptomatology in individuals with FXS, CdLS and RTS. To aid interpretation, the symptomatology of anxiety disorders in these genetic syndromes will be compared to normative data from samples of typically developing children, and children who have received a clinical diagnosis of an anxiety disorder.

## Fragile X Syndrome

FXS affects approximately 1 in 2500–5000 males and 1 in 4000–6000 females (Coffee et al. 2009; Hirst et al. 1993), and is caused by abnormalities in the Fragile X Mental Retardation 1 (FMR1) gene located at Xq27.3, resulting in excessive cytosine–guanine–guanine (CGG) repeats and reduced production of the FMRP protein. As an X-linked disorder, males with the full mutation of FXS are more severely affected than females, and due to these gender differences, males form the focus of this study. A recent meta-analysis indicated that approximately 30% of males with FXS are diagnosed with autism spectrum disorder (ASD; Richards et al. 2015), although a milder profile of autism characteristics is observed than in those with idiopathic autism (Moss et al. 2013).

Individuals with FXS are more likely to meet criteria for an anxiety disorder compared to individuals with Williams syndrome, heterogeneous intellectual disability, and to the general population. Cordeiro et al. (2011) reported that 86.2% of males with FXS met criteria for one anxiety disorder, and 60.3% met criteria for multiple disorders on an informant interview based on DSM-IV criteria. Specific phobia and social phobia were most commonly reported; 64.9 and 60.3% respectively. These phobias occurred more frequently in those meeting criteria for an ASD, but this was not statistically significant. An additional diagnosis of ASD, however, was associated with selective mutism; the third most commonly reported anxiety disorder in males with FXS. The overall figures for anxiety reported by Cordeiro et al. (2011) are similar to those reported by Bailey et al. (2008), in which parental reports demonstrated that 70% of males with FXS had received a formal diagnosis of anxiety or were being treated for anxiety symptoms.

Cordeiro et al. (2011) reported that participants with FXS demonstrated significantly higher rates of anxiety disorder than individuals with idiopathic intellectual disability and individuals with Williams syndrome. This literature highlights the heightened prevalence of anxiety in individuals with FXS compared to that of other syndrome groups and those with idiopathic intellectual disability, suggesting that anxiety is a core phenotypic feature of FXS. The present study aims to contribute to this literature by investigating anxiety in FXS at a symptom-level.

## Cornelia de Lange Syndrome

CdLS affects approximately 1 in 40,000 live births (Beck 1976) and is associated with intellectual disability, as well as specific physical characteristics including distinctive facial features and limb abnormalities. CdLS is caused primarily by a deletion in the NIPBL gene located on chromosome 5 (Gillis et al. 2004; Krantz et al. 2004; Miyake et al. 2005), with fewer cases being caused by mutations on the SMC3 gene on chromosome 10 (Deardorff et al. 2007), the SMC1A gene (Musio et al. 2006), the RAD21 gene (Minor et al. 2014), and the HDAC8 gene (Deardorff et al. 2012). Similarly to FXS, CdLS is associated with an increased risk of ASD with current prevalence estimates around 43% (Richards et al. 2015). In addition, and similar to FXS, individuals show a subtly different profile of autism symptomatology with more impairment in social interaction and communication than in restricted and repetitive behavior (Moss et al. 2013).

Anxiety has been reported in between 10 and 64% of individuals with CdLS (Basile et al. 2007; Berney et al. 1999; Gualtieri 1991; Kline et al. 2007), and existing literature suggests that this increases with age (Basile et al. 2007; Collis et al. 2006; Liu and Krantz 2009) and IQ (Basile et al. 2007). Although anxiety has been reported in CdLS, the symptomatology associated with specific types of anxiety disorder has not yet been extensively investigated in this population.

Social anxiety has been studied independently of other types of anxiety disorder in CdLS and evidence suggests that there may be heightened levels of social anxiety when individuals are presented with particular social demands, such as communication (Richards et al. 2009). Collis et al. (2006) found that behavioral indicators of social anxiety were reported to occur in 75–100% of individuals. This is in agreement with observations of behavioral markers of social anxiety in CdLS, such as being shy and quiet, and high levels of selective mutism (Goodban 1993; Kline et al. 2007; Moss et al. 2016).

## Rubinstein–Taybi Syndrome

RTS is a multiple congenital anomaly syndrome affecting approximately one in 100,000–125,000 live births and is caused by mutations, breakpoints, and microdeletions on chromosome 16p13.3, or by mutations in the E1A Binding Protein, P300, or CREB-binding protein (Hennekam 2006; Lacombe et al. 1992; Petrif et al. 1995; Roelfsema et al. 2005).

Levitas and Reid (1998) reported that 31% of 13 individuals with RTS were diagnosed with a tic/obsessive compulsive disorder. In support of this, Stevens et al. (2011) reported that 31% of individuals with RTS had received a psychiatric diagnosis, and that most of these were for obsessive compulsive disorder, anxiety, or depression. Item-level analysis revealed that 33% of participants were described as having “unreasonable” fears or anxiety. However, the questionnaire used was not standardised or validated. Rather, it consisted of ‘yes or no’ answers to 140 questions about behavior, independence, education and medical problems, limiting the extent to which strong conclusions can be drawn. When comparing anxiety-like symptoms in those with RTS to those without, internalizing behavioral problems including anxiety have been reported to be comparable to a group matched for developmental level, age and gender (Galéra et al. 2009).

Scores on a measure of anxiety/depression were found to be higher in older compared to younger individuals with RTS (Yagihashi et al. 2012). The scores were still in the typical range, although they approached borderline clinical cut-off in the older participants. In contrast, a measure of internalizing, which includes features of anxiety, remained constant over the two age groups. Scores on this subscale were within the borderline clinical cut-off range for older participants with RTS. Item level analyses revealed that 37.5% of younger participants and 64.5% of older participants were reported as ‘too fearful or anxious’. Similarly, 31.3% of the younger participants and 67.7% of the older participants were reported as ‘nervous, high-strung, or tense’. These results suggest that although anxiety is reported in individuals with RTS, it may not be of clinical significance.

Previous research has explored the prevalence of psychiatric disorders in individuals with RTS, which has revealed mixed results. Although these studies provide useful information, the research is limited by small sample sizes and a lack of a comparison groups. Whilst some of these studies have highlighted the presence of anxiety in individuals with RTS, exploration of the symptomatology associated with sub-types of anxiety in this population is warranted.

## Summary and Aims

In summary, evidence suggests the presence of anxiety in individuals with FXS, CdLS and RTS. However, knowledge of the symptoms associated with different types of anxiety in these groups, which is important for targeted interventions, is limited. Furthermore, cross syndrome comparisons of anxiety in these populations have not yet been conducted. These comparisons have the potential to delineate the relationship between anxiety symptomatology, the presence of a particular genetic syndrome, and intellectual disability, which are important factors associated with heightened anxiety. Finally, the phenomenology of anxiety in comparison to individuals with and without diagnosed anxiety disorders has not been extensively explored in these populations.

Measuring anxiety in individuals with an intellectual disability poses a challenge. Tools typically used to assess anxiety include psychiatric interviews, clinical rating scales, and self- and parent-report measures (Bernstein et al. 1996). These often rely on the individual that is experiencing anxiety to self-identify and report symptoms, which may not be possible for people with limited verbal abilities. The diagnosis of anxiety disorders is based on the presence and severity of clusters of symptoms. The present study aims to enhance understanding of anxiety in individuals with FXS, CdLS and RTS by investigating anxiety at this symptom-level. Symptom-based approaches have important implications for assessment, understanding the experiences of an individual with anxiety, and effectiveness of treatments (see Watson 2009 for a review). This is particularly important given the difficulties in applying some elements of the diagnostic criteria of anxiety to individuals with an intellectual disability, and the resultant underdiagnosis (see Cooray and Bakala 2005 for a review).

Data are presented on parental reports of anxiety symptomatology using the Spence Child Anxiety Scale-Parent Version (SCAS-P; Spence 1999), which provides a continuous measure of anxiety symptoms. Due to the heightened prevalence of ASD, particularly in FXS and CdLS, and the potential associations between anxiety and ASD, chronological age and adaptive ability, these associations will be investigated. The anxiety disorders investigated in the current study are not inclusive of every disorder listed in the DSM-5 but do include separation anxiety, generalized anxiety disorder, panic attack and agoraphobia, physical injury fears, social phobia and obsessive–compulsive disorder.

The SCAS-P was designed as a parental report measure to assess anxiety symptomatology in children. Although the present study investigates symptoms of anxiety in children *and adults* with FXS, CdLS and RTS, a parent-report measure designed for children was deemed most appropriate due to the severity and range of intellectual disability in these populations. Self-report data, particularly in combination with other measures, is a valuable source of information. However, due to the reliance on understanding and labeling emotions for accurate self-report of anxiety, alongside heightened levels of acquiescence in individuals with an intellectual disability (Stancliffe 2000), and the severity of intellectual disability of participants in the current study, a parental measure was most likely to yield robust data in this case.

The data obtained from participants with FXS, CdLS and RTS were compared to normative data from typically developing participants and normative data from participants diagnosed with an anxiety disorder (Nauta et al. 2004). Although it was not possible to match the normative participant samples to the participants with FXS, CdLS and RTS due to the rarity of the genetic syndrome groups, the normative data serve as a benchmark for comparison, which aids interpretation. As existing literature on anxiety in these populations is limited, these data provide a strong starting point for further research questions and studies to be developed.

In this study, we address three primary research questions: (1) How do individuals with FXS, CdLS and RTS differ in their profile of anxiety symptomatology? (2) What is the relationship between anxiety and chronological age, and anxiety and ASD? (3) How do individuals with FXS, CdLS and RTS differ in anxiety symptomatology to typically developing children and individuals diagnosed with anxiety?

## Method

### Participants

Parents of 19 individuals with FXS (0 female, M_age_ = 24.19, SD 7.51), 13 individuals with CdLS (7 female, M_age_ = 18.75, SD 9.75), and 27 individuals with RTS (17 female, M_age_ = 23.55, SD 10.74) completed the measures for this study. Participants with FXS were recruited through the database held at the Cerebra Centre for Neurodevelopmental Disorders, University of Birmingham. Participants with CdLS and RTS were recruited through the Cornelia de Lange Foundation (UK and Ireland), and the Rubinstein–Taybi Syndrome UK Support Group, respectively. All participants had previously received a diagnosis of FXS, CdLS, or RTS from a pediatrician or clinical geneticist. Participant characteristics are presented in Table 1. Due to documented gender differences in FXS, all participants with FXS were male. Therefore, participants are not matched on sex. However, the three participant groups are comparable for chronological age, global adaptive behavior, verbal adaptive behavior, and severity of autism symptomatology.

**Table 1:** Participant characteristics and comparison statistics for participants with FXS, CdLS and RTS

	Fragile X	Cornelia de Lange	Rubinstein–Taybi	*p* value for comparison
	(n = 19)	(n = 13)	(n = 27)	
Chronological age (SD)	24.19 (7.51)	18.75 (9.75)	23.55 (10.74)	0.247
Gender % male	100.00	46.15	37.04	<0.001
Adaptive behavior composite standard score mean (SD)	46.05 (16.67)	50.08 (17.50)	43.59 (16.51)	0.522
Verbal adaptive behavior standard score mean (SD)	38.47 (19.34)	47.23 (22.02)	42.30 (18.78)	0.470
SCQ total score	17.99 (6.61)	19.60 (6.56)	17.76 (6.06)	0.679

Normative data on typically developing individuals with and without a diagnosed anxiety disorder (obtained from Nauta et al. 2004) are used in the present study. These normative data are from 261 typical controls aged 6–18 years (M_age_ = 11.5, SD 2.0) and 484 children diagnosed with an anxiety disorder aged 6–17 years (M_age_ = 10.4, SD 2.5).

### Measures

The following measures were completed by the participant’s primary caregiver.

#### Vineland Adaptive Behavior Scale—Second Edition, Survey Interview Form (VABS; Sparrow et al. 2005)

This semi-structured interview was administered to primary caregivers of participants with FXS, CdLS and RTS, in order to assess adaptive behavior in the areas of communication, daily living skills and socialization. The interview yields an Adaptive Behavior Composite (ABC) and standard scores based on a sample of 3000 children.

#### Social Communication Questionnaire (SCQ; Rutter et al. 2003)

The SCQ is an informant questionnaire designed to assess behaviors associated with ASD such as repetitive behavior, communication and social interaction. The authors of the SCQ suggest that a score of 15 or above indicates the presence of ASD, whereas a score of 22 or above indicates the presence of autism. Internal consistency and concurrent validity with the Autism Diagnostic Observation Schedule (Lord et al. 1999) are good (Howlin and Karpf 2004).

#### The Spence Child Anxiety Scale—Parent Version (Spence 1999)

The SCAS-P is a 38-item informant questionnaire designed to assess anxiety symptoms in children. The SCAS-P assesses symptomatology associated with the following six domains of anxiety: physical injury fears, obsessive–compulsive disorder, separation anxiety, social phobia, panic/agoraphobia, and generalized anxiety. Psychometric properties show the scale to significantly differentiate those with and without an anxiety disorder (Nauta et al. 2004). In addition, when used with children and adolescents with ASD, the SCAS-P demonstrates good to excellent internal consistency for the total score, acceptable to good internal consistency at a subscale level, and convergent validity with the Development Behavior Checklist-Parent Version total score and anxiety subscale (Einfeld and Tongue 2002; Zainal et al. 2014). Zainal et al. (2014) suggested that psychometric properties of the SCAS-P are similar when the measure is used with children and adolescents with and without ASD, based on data reported by Nauta et al. (2004), and that a diagnosis of a neurodevelopmental disorder such as ASD does not appear to compromise the validity of the measure. The measure has also been described as robust for use in the ASD population (Wigham and McConachie 2014).

### Procedure

The measures in this study were included in a questionnaire pack given to parents and caregivers of participants taking part in a larger study on cognitive and social difficulties in FXS, CdLS and RTS (Crawford et al. 2016). Parents and carers of participants were given the questionnaire pack either at a syndrome support group meeting or during a research visit. The questionnaires were either returned on the same day or via post following the meeting or visit. The VABS was either administered during the research visit or over the phone following the meeting or visit.

### Data Analysis

All data were subjected to the Shapiro–Wilk test for normality. Non-parametric tests were used to confirm results from parametric tests for data that were not normally distributed. For consistency, results from parametric tests are reported where non-parametric tests revealed the same results. Except where mentioned, the alpha level for significance was 0.05.

## Results

**Fig. 1:**
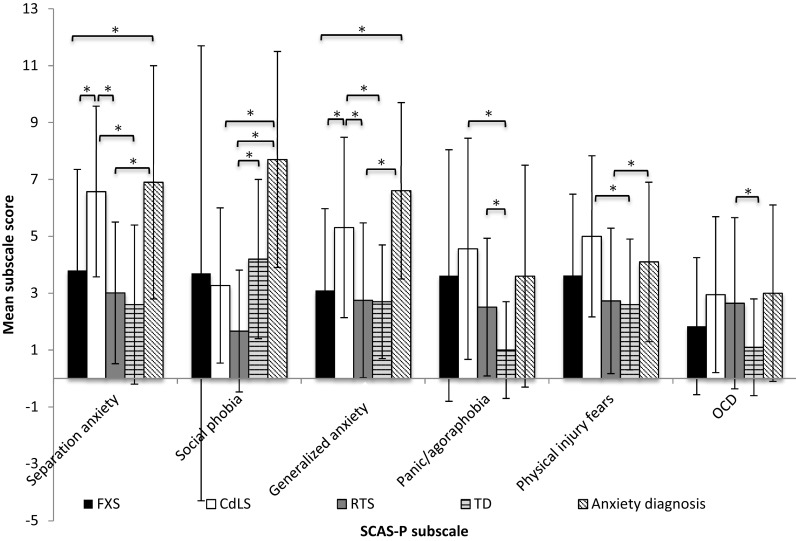
The mean scores on each subscale of the SCAS-P for each syndrome group and two normative comparison groups. *Error bars* represent standard deviation, as standard error data were not available for normative data

Figure 1 displays the mean scores on each subscale for participants with FXS, CdLS and RTS, as well as the mean scores from normative data on typically developing children and children with an anxiety disorder. The total scores on the SCAS-P were: FXS: Mean = 19.71, SD 16.50; CdLS: Mean = 27.66, SD 13.08; RTS: Mean = 15.32, SD 11.05. The total scores on the SCAS-P from normative data are: typically developing children: Mean = 14.2, SD 9.7; anxiety disorder: Mean = 31.8; SD 14.1 (Nauta et al. 2004). The percentage of participants scoring outside of the normal range, as defined by the mean + 1 standard deviation using the national normal data, is displayed in Table 2.

**Table 2:** Summary of comparisons between syndrome groups and normative data on subscales of the SCAS-P (< group at the top of the table scored lower than the group listed in the column, = no difference between scores, > group at the top of the table scored higher than the group listed in the column)

	Fragile X (FXS)	Cornelia de Lange (CdLS)	Rubinstein–Taybi (RTS)
Separation anxiety	<**CdLS**	>**FXS**	=FXS
	=RTS	>**RTS**	<**CdLS**
	=TD	>**TD**	=TD
	<**AD**	=AD	<**AD**
Percentage of participants scoring above normal range	26.3%	61.5% (50% of males, 71.4% of females)	14.8% (20% of males, 11.8% of females)
Social phobia	=CdLS	=FXS	=FXS
	=RTS	=RTS	=CdLS
	=TD	=TD	<**TD**
	=AD	<**AD**	<**AD**
Percentage of participants scoring above normal range	15.8%	7.7% (16.7% of males, 0% of females)	3.7% (10% of males, 0% of females)
Generalized anxiety	<**CdLS**	>**FXS**	=FXS
	=RTS	>**RTS**	<**CdLS**
	=TD	>**TD**	=TD
	<**AD**	=AD	<**AD**
Percentage of participants scoring above normal range	26.3%	61.5% (33.3% of males, 85.7% of females)	18.5% (30% of males, 11.8% of females)
Trajectory		+**Age** ^**a**^	
Panic/Agoraphobia	=CdLS	=FXS	=FXS
	=RTS	=RTS	=CdLS
	>**TD** ^a^	>**TD**	>**TD**
	=AD	=AD	=AD
Percentage of participants scoring above normal range	47.4%	53.8% (50% of males, 57.1% of females)	37% (50% of males, 29.4% of females)
Trajectory		+**Age**	
Physical injury fears	=CdLS	=FXS	=FXS
	=RTS	=RTS	=CdLS
	=TD	>**TD**	=TD
	=AD	=AD	<**AD**
Percentage of participants scoring above normal range	36.8%	46.2% (33.3% of males, 57.1% of females)	22.2% (40% of males, 11.8% of females)
Obsessive–compulsive disorder	=CdLS	=FXS	=FXS
	=RTS	=RTS	=CdLS
	=TD	=TD	>**TD**
	=AD	=AD	=AD
Percentage of participants scoring above normal range	36.8%	38.5% (33.3% of males, 42.9% of females)	40.7% (60% of males, 29.4% of females)
Trajectory	+**Age** **−Adaptive behavior**	+**Age**	
Total score	=CdLS	=FXS	=FXS
	=RTS	>**RTS**	<**CdLS**
	=TD	>**TD**	=TD
	<**AD**	=AD	<**AD**
Percentage of participants scoring above normal range	36.8%	53.8% (50% of males, 57.1% of females)	18.5% (30% of males, 11.8% of females)

Significant associations between subscale scores and participant characteristics (+ positive correlation; − negative correlation), and the percentage of participants scoring above the normal range, are also noted. Significant differences are indicated in bold

*TD* typically developing, *AD* anxiety diagnosis

^a^Marginally significant result

### Syndrome Group Comparison

One-way ANOVAs revealed a significant between-groups difference in the following subscales of the SCAS-P: separation anxiety (*F* (2, 58) = 6.379, *p* = 0.003), generalized anxiety (*F* (2, 58) = 3.667, *p* = 0.032), and total score (*F* (2, 58) = 3.697, *p* = 0.031). There were no between-groups differences in the subscales assessing panic attack and agoraphobia, physical injury fears, social phobia, and obsessive–compulsive disorder (*p* > 0.05). Bonferroni post-hoc analyses revealed that differences in the separation anxiety subscale were due to participants with CdLS scoring higher than participants with FXS (*p* = 0.038) and RTS (*p* = 0.002). Differences in the generalized anxiety subscale were also due to participants with CdLS scoring higher than participants with FXS (*p* = 0.033) and participants with RTS (*p* = 0.006). Finally, differences in the total score were due to participants with CdLS scoring higher than participants with RTS (*p* = 0.026).

### Relationship Between Anxiety, Chronological Age and Autism Symptomatology

**Fig. 2:**
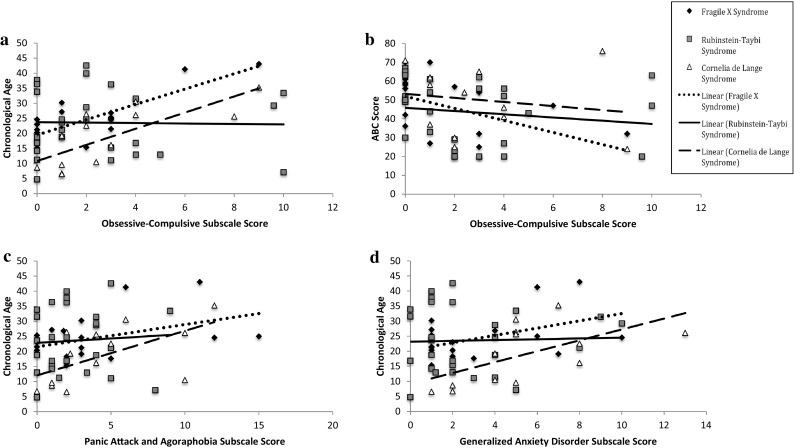
The relationship between participant characteristics and the SCAS-P for each syndrome group

Spearman’s rho correlational analyses were conducted for each group to investigate whether there was a relationship between any subscale on the SCAS-P and chronological age, global adaptive behavior, verbal adaptive behavior and autism symptomatology. The alpha level was adjusted to 0.01 to account for multiple correlations. Significant correlations are depicted in Fig. 2.

For the FXS group, the analyses revealed a positive relationship between scores on the obsessive–compulsive subscale and chronological age (Fig. 2a; r_s_ (17) = 0.643, *p* = 0.003), and a negative relationship between scores on the obsessive–compulsive subscale and global adaptive behavior (Fig. 2b; r_s_ (17) = −0.597, *p* = 0.007). For the CdLS group, significant positive relationships were revealed between chronological age and scores on the panic attack and agoraphobia subscale (Fig. 2c; r_s_ (11) = 0.688, *p* = 0.009), and the obsessive–compulsive subscale (Fig. 2a; r_s_ (11) = 0.785, *p* = 0.001), and a marginally significant positive relationship was revealed between chronological age and scores on the generalized anxiety disorder subscale (Fig. 2d; r_s_ (11) = 0.680, *p* = 0.011). There were no significant correlations between the subscales of the SCAS-P and participant characteristics for the RTS group.

### Comparison to Normative Data

One sample *t* tests were carried out using the mean and standard deviation on each subscale of the SCAS-P for each syndrome group in order to compare their data to normative data for typically developing individuals with and without a diagnosed anxiety disorder (obtained from Nauta et al. 2004). For children with an anxiety disorder, means and standard deviations on each subscale of the SCAS-P are also available by the type of anxiety disorder that they have been diagnosed with (separation anxiety, generalized anxiety, social phobia, panic/agoraphobia, specific phobia and OCD). Where possible, for any subscale in which participants with FXS, CdLS or RTS scored similarly to the group of children with an anxiety disorder, further comparisons were made to investigate whether scores remained similar when compared to children diagnosed with the sub-type of anxiety reflected in the subscale of the SCAS-P. For example, if participants with FXS did not score differently to the children with an anxiety disorder on the social phobia subscale of the SCAS-P, their scores were then compared to scores of children diagnosed with social phobia. As a specific type of anxiety disorder does not represent the physical injury fears subscale, the above comparisons were not conducted when there were no differences between participants with FXS, CdLS or RTS and children with an anxiety disorder on the physical injury fears subscale. Due to multiple analyses, the *p*-value was adjusted to 0.01.

### Fragile X Syndrome

In comparison to normative data collected from typically developing participants, participants with FXS only scored marginally higher on the panic/agoraphobia subscale (*t* (279) = 2.570, *p* = 0.01). On all other subscales, no significant differences were revealed between participants with FXS and normative data collected from typically developing participants (all *p* > 0.01).

Scores did not differ between children diagnosed with an anxiety disorder and participants with FXS on the social phobia subscale (*t* (502) = −2.170, *p* = 0.03), the panic attack/agoraphobia subscale (*t* (502) = 0.019, *p* = 0.985), the physical injury fears subscale (*t* (502) = −0.706, *p* = 0.481), and the obsessive–compulsive disorder subscale (*t* (502) = −2.033, *p* = 0.043). For all other subscales and the total score, participants with FXS scored significantly lower than the children diagnosed with an anxiety disorder (all *p* < 0.01).

### Cornelia de Lange Syndrome

Participants with CdLS scored higher than a normative sample of typically developing children on the separation anxiety subscale (*t* (273) = 4.671, *p* < .001), the generalized anxiety subscale (*t* (273) = 2.940, *p* = 0.004), the panic/agoraphobia subscale (*t* (273) = 3.284, *p* = 0.001), the physical injury fears subscale (*t* (273) = 3.009, *p* = 0.003), and on the total score (*t* (273) = 3.677, *p* < 0.001). No differences were revealed between participants with CdLS and the normative sample of typically developing children on the social phobia subscale and the obsessive–compulsive disorder subscale (all *p* < 0.01).

No differences were revealed between participants with CdLS and children diagnosed with an anxiety disorder on any subscale (all *p* > 0.01), except on the social phobia subscale, where participants with CdLS scored significantly lower than children with an anxiety disorder (*t* (495) = −0.704, *p* < 0.001). No differences were revealed between participants with CdLS and children diagnosed with generalized anxiety disorder on the generalized anxiety subscale (*t* (175) = −1.411, *p* = 0.160).

### Rubinstein–Taybi Syndrome

Participants with RTS scored significantly lower than the normative typically developing sample on the social phobia subscale (*t* (287) = −5.662, *p* < 0.001), and significantly higher than typically developing children on both the panic and agoraphobia subscale (*t* (287) = 3.163, *p* = 0.002) and the OCD subscale (*t* (287) = 2.633, *p* = 0.009). Scores between typically developing participants and participants with RTS did not differ on the other subscales of separation anxiety, generalized anxiety, physical injury fears, and the total score (all *p* > 0.01).

Participants with RTS did not evidence significantly different scores to children with an anxiety disorder on the subscales of panic/agoraphobia and obsessive–compulsive disorder (*t* (509) = −0.587, *p* = 0.557), but they did evidence lower scores than children diagnosed with obsessive–compulsive disorder (*t* (43) = −5.337, *p* < 0.001). For all other subscales, participants with RTS scored significantly lower than the children diagnosed with an anxiety disorder (all *p* < 0.01). The results from the comparative and correlational analyses are presented in Table 2.

## Discussion

This study conducted subscale level analysis using the SCAS-P to identify similarities and differences in the profile of anxiety symptomatology in individuals with FXS, CdLS and RTS. The relationship between anxiety and participant characteristics was also examined. In addition, subscale scores on the SCAS-P were compared to normative data available from typically developing children, children diagnosed with an anxiety disorder, and, where possible, children diagnosed with the specific anxiety disorder reflected in each subscale of the informant report measure.

To summarise, participants with CdLS scored higher than participants with FXS and RTS on the separation anxiety and generalized anxiety subscales of the SCAS-P. In addition, there were no differences between participants with FXS and children diagnosed with an anxiety disorder on the social phobia, panic attack/agoraphobia, obsessive-compulsive disorder, or the physical injury fears subscales. There were also no differences between participants with RTS and children diagnosed with an anxiety disorder on the panic attack/agoraphobia and obsessive–compulsive disorder subscales. Finally, there were no differences between participants with CdLS and children diagnosed with an anxiety disorder on the separation anxiety, generalized anxiety, panic attack/agoraphobia, physical injury and obsessive–compulsive disorder subscales, and on the total score. When compared to children diagnosed with specific types of anxiety disorder reflected in the subscales of the SCAS-P, only the scores from participants with CdLS on the generalized anxiety subscale could not be differentiated from those diagnosed with generalized anxiety disorder. It is important to note that the genetic syndrome groups were not matched to the typically developing children and/or children diagnosed with an anxiety disorder. Therefore, these normative data should be viewed as a benchmark comparison to aid interpretation of the results from the genetic syndrome groups.

Positive associations were reported between the chronological age of participants with CdLS and their scores on the generalized anxiety, panic attack/agoraphobia and obsessive–compulsive disorder subscales, supporting existing literature indicating that heightened anxiety may coincide with increasing age in this group (Basile et al. 2007; Collis et al. 2006). The associations between anxiety and chronological age were not seen in the RTS group and only emerged in the FXS group on the obsessive–compulsive disorder subscale. In addition, there was no relationship between autism symptomatology and anxiety for any participant group. This supports previous research indicating no difference in anxiety symptomatology in participants with FXS and an additional diagnosis of ASD compared to those without an additional diagnosis (Cordeiro et al. 2011).

The findings of this study support previous literature demonstrating elevated levels of social phobia, panic disorder with agoraphobia, and obsessive–compulsive disorder in individuals with FXS compared to a matched comparison group (Cordeiro et al. 2011), and extends this by suggesting the severity of symptomatology of these disorders are similar in FXS to those seen in individuals diagnosed with an anxiety disorder. In addition, heightened levels of specific phobia have been reported in FXS (Cordeiro et al. 2011). Whilst the measure utilized in the present study did not assess specific phobia, it did demonstrate elevated scores on the ‘physical injury fears’ subscale, which may be considered a specific phobia. Previous research has also demonstrated higher rates of generalized anxiety disorder in FXS (Cordeiro et al. 2011), a finding not replicated in the present study. A potential reason for these contrasting findings is the utilization of different methodologies to study anxiety diagnosis versus anxiety symptomatology. For example, in the present study, a parent-report measure was used to delineate the profile and investigate the symptomatology of different subtypes of anxiety, whereas the study conducted by Cordeiro et al. (2011) investigated the prevalence rates of individuals with FXS meeting diagnostic criteria for a range of anxiety disorders. It is important to note that, in the present study, the FXS group did not score higher on any subscales of the measure used than the other two genetic syndrome groups matched for adaptive behavior and chronological age. Therefore, the higher levels of social phobia, panic with agoraphobia, physical injury fears, and obsessive–compulsive disorder may be related to developmental ability, although the comparison groups have also been associated with anxiety both in the present study and in previous literature.

The findings reported here document the severity and breadth of anxiety symptomatology in individuals with CdLS. In particular, individuals with CdLS demonstrated higher levels of generalized anxiety disorder and separation anxiety than both typically developing children and individuals with different genetic syndromes associated with intellectual disability. For these two types of anxiety disorder, no differences were revealed between individuals with CdLS and those diagnosed with an anxiety disorder, highlighting the severity of anxiety disorders in this population. Aside from social phobia, participants with CdLS demonstrated similar levels of all other types of anxiety disorders measured here to normative data obtained from individuals diagnosed with an anxiety disorder, thus highlighting the broad nature of anxiety disorder in this group. The results from the present study support previous literature suggesting obsessive–compulsive features in individuals with CdLS (Kline et al. 2007), and extends this to indicate the range of additional anxiety disorders present in this group.

In the present study, individuals with RTS did not demonstrate heightened levels of anxiety in comparison to the other genetic syndrome groups. However, levels of panic attack/agoraphobia and obsessive–compulsive disorder were reported to be higher in RTS than typically developing children, and similar to those diagnosed with an anxiety disorder. This supports previous literature highlighting the presence of obsessive–compulsive features in this population (Levitas and Reid 1998; Stevens et al. 2011). It is important to note that repetitive behavior is a well-documented characteristic of RTS (Waite et al. 2015). Therefore, the elevated scores on the obsessive–compulsive disorder subscale may reflect heightened levels of repetitive behavior, rather than the full range of symptomatology associated with obsessive-compulsive disorder.

The results reported here highlight that different types of anxiety disorder may be problematic for individuals with different genetic syndromes. However, it is possible that whilst there may be similarities in the ways in which these anxiety disorders manifest behaviorally in individuals with genetic syndromes and the general population, differences may exist between these populations in the mechanisms subserving these behaviors. This notion is supported by the conceptual differences in anxiety that have been reported between individuals with and without ASD (Kerns et al. 2014). In particular, it may be the case that ASD-related impairments, such as adherence to routine, may contribute to the anxiety in individuals with ASD that are not consistent with anxiety diagnostic categories such as intolerance to uncertainty (Rodgers et al. 2016). In the current study, elevated levels of panic attack and agoraphobia symptomatology in individuals with FXS may be related to hypersensitivity of sensory stimuli, a common feature of this genetic syndrome (Baranek et al. 2009; Cornish et al. 2008). This behavioral characteristic may contribute to anxiety in settings where sensory input is heightened, such as crowded places like shopping centers and busy playgrounds. The presence of anxiety in crowded places features as an item of the panic attack and agoraphobia subscale of the SCAS-P. Further understanding the underlying mechanisms of anxiety disorder in neurodevelopmental disorders is paramount to developing targeted interventions.

The results of the present study should be considered in light of some limitations. First, whilst the genetic syndrome groups were statistically comparable on chronological age, these groups were not matched to the samples from which normative data were obtained. Therefore, comparisons between the syndrome groups and normative data groups should be treated with caution and future research should conduct comparisons with matched groups. Second, the SCAS-P was designed to measure anxiety symptomatology in young, typically developing children. There is a lack of suitable measures of anxiety that are designed for children and adults with an intellectual disability and further research is required to develop such measures. Despite the limitations, this study conducted an initial investigation into the profile of anxiety disorder and has reported elevated levels of symptoms associated with different sub-types of anxiety in individuals with three different genetic syndromes. This requires further investigation using diagnostic parent report measures alongside behavioral observation and clinical consultation. Extrapolating these phenotypic differences is crucial to further understand the nature and severity of anxiety that people with these genetic syndromes experience.
